# Beam propagation management in a fractional Schrödinger equation

**DOI:** 10.1038/s41598-017-05926-5

**Published:** 2017-07-14

**Authors:** Changming Huang, Liangwei Dong

**Affiliations:** Department of Physics, Shaanxi University of Science & Technology, Xi’an, 710021 China

## Abstract

Generalization of Fractional Schrödinger equation (FSE) into optics is fundamentally important, since optics usually provides a fertile ground where FSE-related phenomena can be effectively observed. Beam propagation management is a topic of considerable interest in the field of optics. Here, we put forward a simple scheme for the realization of propagation management of light beams by introducing a double-barrier potential into the FSE. Transmission, partial transmission/reflection, and total reflection of light fields can be controlled by varying the potential depth. Oblique input beams with arbitrary distributions obey the same propagation dynamics. Some unique properties, including strong self-healing ability, high capacity of resisting disturbance, beam reshaping, and Goos-Hänchen-like shift are revealed. Theoretical analysis results are qualitatively in agreements with the numerical findings. This work opens up new possibilities for beam management and can be generalized into other fields involving fractional effects.

## Introduction

Recently, there have been widespread efforts in the field of beam propagation management, motivated mainly by its fundamental interests and potential applications in all-optical steering, switching, and routing^1–19^. Most of them are based on the periodic modulation of optical materials along the transversal/longitudinal directions. Many interesting phenomena have been reported, such as nondiffractive propagation of light beams in zigzag waveguide arrays^1^, structure photonic crystals^2^ and Kapitza media^3^, Rabi oscillations and periodic shape transformations^4, 5^, resonant suppression of light coupling^6–9^, dragging of laser beams^10^, diffraction-managed solitons^11, 12^, linear and nonlinear unidirectional edge states^13–16^, and all-optical steering and switching^17–19^, just to name a few.

Another issue attracting growing attentions these days is the fractional effect occurring in various areas of physics, e.g., Quantum Hall effect^20^, Talbot effect^21^, Josephson effect^22^, and quantum oscillator^23^. Laskin generalized the standard Schrödinger equation (SE) into the FSE by replacing the second-order spatial derivative with a fractional Lévy index^24–26^. FSE describes the behavior of particles with fractional spin and thus takes into account of fractional effect in quantum mechanics.

In 2015, Longhi generalized the FSE into optics by considering the perfect analogue between the SE and the paraxial wave equation^27^. Dual-Airy states were predicted and the relevant optical implementation was suggested. This work opened a new area of beam dynamics in the FSE and inspired several intriguing studies on the beam propagation in both linear and nonlinear configurations^28–34^.

Following^27^, Zhang *et al*. investigated the beam dynamics in the FSE with or without an external potential and found series of fascinating features, including the zigzag propagation of chirped Gaussian beam in a parabolic potential^28^, conical diffraction in ${\mathscr{P}}{\mathscr{T}}$-symmetry periodic lattices^29^, diffraction-free beams in uniform media^30^, and linear modes trapped in a harmonic-oscillator potential^31, 32^. Note that the experimental setting for the realization of beam propagation in the FSE was proposed very recently^30^. In the context of nonlinearity, nonlinear effects tuned by varying the Lévy index^33^ and stable lattice solitons in focusing/defocusing Kerr media^34^ have been uncovered.

Considering the fact that fractional effect can effectively suppress the beam diffraction and the interaction between a beam and a refractive-index potential can be used to control the behaviour of beam evolution, we suggest a simple model for the realization of propagation management. A double-barrier potential including two waveguides featuring a Gaussian distribution is introduced into the FSE. We reveal that, for fixed Lévy index close to 1, there exist two critical potential depths (*p*_cr1_ and *p*_cr2_). Below *p*_cr1_, an oblique input transmits the potential straightly. Above *p*_cr2_, an oblique input undergoes totally reflection and follows a zigzag trajectory, analogous to ref. 28. When *p* ∈ [*p*_cr1_, *p*_cr2_], a beam can be partially transmitted and partially reflected. It provides a powerful and effective way for the beam management and can find many applications in optics, such as switching, routing, and reshaping.

## Theoretical model

We consider beam propagation along the *ξ* axis in linearly uniform media with an external potential. Its dynamics is governed by the fractional Schrödinger equation^27^:

1 $$i\frac{\partial {\rm{\Psi }}(\eta ,\xi )}{\partial \xi }=\frac{1}{2}{(-\frac{{\partial }^{2}}{\partial {\eta }^{2}})}^{\alpha \mathrm{/2}}{\rm{\Psi }}(\eta ,\xi )+V(\eta ){\rm{\Psi }}(\eta ,\xi \mathrm{)}.$$

Here, Ψ is the dimensionless field amplitude, *η* and *ξ* are the normalized transverse and longitudinal coordinates, respectively. Parameter *α* is the so-called Lévy index satisfying the condition 1 < *α* ≤ 2. It describes the fractional-order diffraction effect. When *α* = 2, Eq. (1) degenerates to the standard SE. With the decrease of Lévy index, the diffraction rate of a beam becomes weak^34^. The function *V*(*η*) is a refractive-index potential which can be designed as diverse forms. Numerically, Eq. (1) can be solved by using the split-step Fourier method. In the one-dimensional case, (−Δ)^*α*/2^ *f* can be defined as $\widehat{{(-{\rm{\Delta }})}^{\alpha \mathrm{/2}}f}(k)={|k|}^{\alpha }\hat{f}(k)$ ^35^.

To realize the propagation control of light beams, we consider a double-barrier potential in the form of $V(\eta )=p\{\exp [-{(\eta -{\eta }_{0})}^{2}/{d}_{0}^{2}]+\exp [-{(\eta +{\eta }_{0})}^{2}/{d}_{0}^{2}]\}$. Without loss of generality, we fix *η*_0_ = 10, *d*_0_ = 1 and vary the potential depth *p*. The input is assumed as a modulated Gaussian beam:

2 $${\rm{\Psi }}(\eta ,\xi =\mathrm{0)}=A\exp (-{\eta }^{2}/{d}^{2})\cos (\gamma \eta )\exp (-i\kappa \eta ),$$

where *d* is the beam width, *κ* denotes the transverse wavenumber, and *γ* is a modulation frequency. *A*, *d* = 1 unless stated elsewhere. We define the central coordinate ${\eta }_{c}={\int }_{-\infty }^{+\infty }{|{\rm{\Psi }}|}^{2}\eta d\eta /{\int }_{-\infty }^{+\infty }{|{\rm{\Psi }}|}^{2}d\eta$ and the integral form-factor $\chi ={\int }_{-\infty }^{+\infty }{|{\rm{\Psi }}|}^{4}d\eta /{[{\int }_{-\infty }^{+\infty }{|{\rm{\Psi }}|}^{2}d\eta ]}^{2}$, which manifest the propagation trajectory and the localization degree, respectively.

## Numerical results and discussions

### Propagation of Gaussian beams

**Figure 1:**
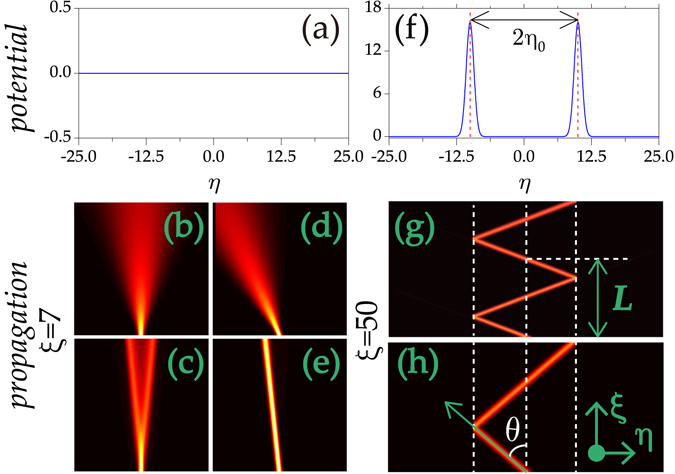
Evolution of beams in uniform media with and without a double-barrier modulation. (**a**) Uniform medium. (**f**) Double-barrier structure. The evolution of Gaussian beams with *κ* = 0 (**b**,**c**) and 3 (**d**,**e**) in uniform media. *α* = 2 in (**b**,**d**) and 1.2 in (**c**,**e**). (**g**,**h**) Propagation of Gaussian beams with *κ* = 50. *α* = 1.2, *p* = 90 in (**g**) and *α* = 1.02, *p* = 40 in (**h**).

First, we address the simplest case when no potential is presented [Fig. 1(a)]. For Lévy index *α* = 2 (SE), the vertical incident beam with *κ* = 0 and *γ* = 0 experiences a natural diffraction [Fig. 1(b)]. The oblique Gaussian beam exhibits a similar behaviour [Fig. 1(d)].

Yet, for small Lévy index, e.g., *α* = 1.2, the normal incidence splits into two oblique parts after a short distance [Fig. 1(c)]. Nevertheless, an oblique incidence whose diffraction rate depends on the Lévy index always propagates along the direction of the initial input [Fig. 1(e)]. This property is very important and remarkably different from the cases in the SE. It provides a prerequisite for the beam propagation management. Similar analogues were discussed in the extreme limit at *α* = 1^30^.

The tilted input beam encounters one of the double barriers at *η* = ±*η*_0_ [Fig. 1(f)]. To study the beam propagation over a long distance, we assume the Lévy index close to 1 hereinafter. For deep potential, the field is completely confined in the region between two barriers and propagates along a periodical zigzag trajectory [Fig. 1(g,h)]. This phenomenon is similar to the “mirror reflection” or “total reflection” in classical optics. The longitudinal periodicity $L=4{\eta }_{0}\,\tan (\theta )$ with *θ* depicting the propagation direction. The slight variation of Lévy index can change the propagation periodicity obviously.

**Figure 2:**
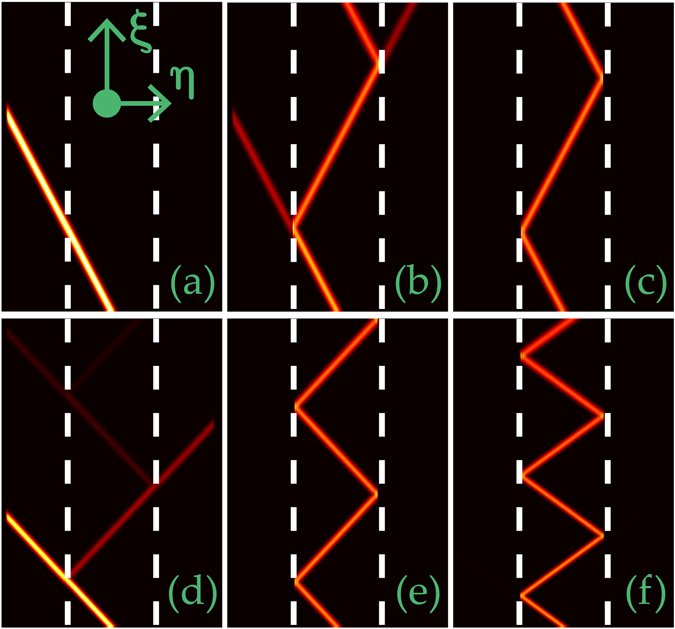
Propagation path management of light beams by potential depth. Propagation of an oblique Gaussian beam in a double-barrier potential with *p* = 20 (**a**), 36 (**b**), 60 (**c**,**d**), 95 (**e**), and 140 (**f**), respectively. *α* = 1.08 in (**a**–**c**), 1.2 in (**d**,**e**) and 1.28 in (**f**). In all the plots, *κ* = 50, *η* ∈ [−25, 25], and *ξ* ∈ [0, 50].

The propagation dynamics of beams can be classified into three regimes. When the potential is shallow, e.g., *p* = 20, a beam passes the barrier without any distortions [Fig. 2(a)]. It does not “feel” the existence of potential and thus is in contrast to all reported related phenomena. At moderate barrier height (*p* = 36), both transmission and reflection occur simultaneously [Fig. 2(b)]. If the potential is deep enough, beams always experience a total reflection [Fig. 2(c)]. Notice that the periodicity is independent on the potential depth. We thus draw an important conclusion that the variation of potential depth offers an effective and convenient way for the beam management in the FSE.

While a beam experiences a total reflection at *α* = 1.08 [Fig. 2(c)], it undergoes a partial transmission and a partial reflection at *α* = 1.2 for the same potential depth [Fig. 2(d)]. For larger Lévy index, one needs even higher potential to grantee the total reflection [Fig. 2(e)]. Further increase of Lévy index leads to the decrease of propagation periodicity [Fig. 2(f)].

**Figure 3:**
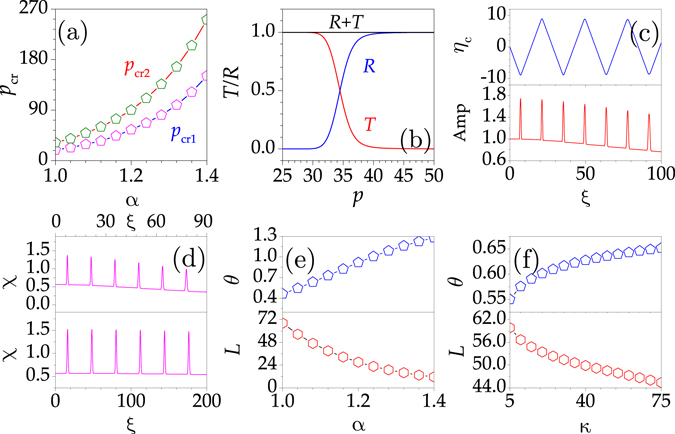
Propagation properties of Gaussian beams in the FSE. (**a**) Lower and upper critical potential depths versus the Lévy index *α*. (**b**) Dependent of reflectivity *R* and transmissivity *T* on depth *p*. (**c**) Propagation trajectory (top) and the variation of amplitude (bottom). (**d**) Integral form-factor *χ* for *α* = 1.2 (top) and 1.02 (bottom) versus *ξ*. The output angle *θ* (top) and longitudinal periodicity *L* (bottom) versus *α* for fixed *κ* (**e**) and versus *κ* for fixed *α* (**f**). Parameters: *α* = 1.08 in (**b**) and 1.08 in (**c**). *p* = 100 in (**c**,**d**), and *κ* = 50 in (**a**–**e**).

The properties of beam propagation in the FSE with two Gaussian potentials are summarized in Fig. 3. The dependent of critical depths on the Lévy index is shown in Fig. 3(a). Beyond the upper critical depth, a beam is totally reflected. Below the lower critical depth, a beam propagates freely. When *p* ∈ [*p*_cr1_, *p*_cr2_], a beam can be transmitted and reflected simultaneously. The attenuation rate of beam energy is determined by the potential depth. These features are clearly demonstrated by the reflectance and transmittance curves shown in Fig. 3(b).

The propagation trajectory of a beam characterized by its central coordinate *η*_*c*_ is illustrated in Fig. 3(c). The beam propagates along a zigzag path due to the total reflection induced by the deep potential. However, the beam suffers a weak diffraction, since the Lévy index is larger than 1. It leads to the slow decrease of peak intensity upon evolution [Fig. 3(c)]. The peak value increases abruptly when the beam encounters a barrier. It means that the beam undergoes an obvious deformation. The beam recovers its original distribution once it leaves the potential, which indicates that the beam in such a system has a strong self-healing ability.

At *α* = 1.2, the input beam becomes broader because the integral form-factor *χ* decreases with *ξ* [Fig. 3(d)]. The diffraction effect can be ignored when *α* → 1 [bottom plot in Fig. 3(d)]. For fixed transverse wavenumber *κ* (Lévy index *α*), the propagation angle increases monotonously with the growth of *α* (*κ*) [top plots in Fig. 3(e,f)]. Meanwhile, the longitudinal periodicity decreases with *α* (*κ*) [bottom plots in Fig. 3(e,f)]. Therefore, one can also control the propagation of beams by varying the Lévy index of the FSE and the transverse wavenumber of the initial input.

### Propagation of complex beams

**Figure 4:**
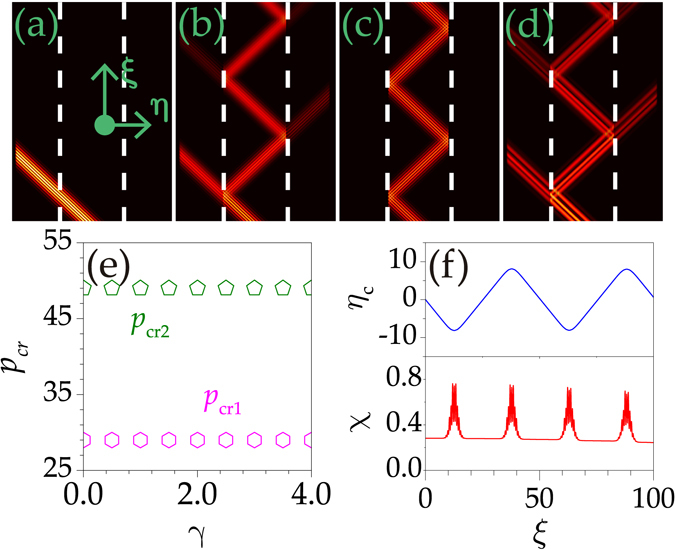
Evolution of complex light beams in potentials with depth *p* = 20 (**a**), *p* = 35 (**b**,**d**), and *p* = 55 (**c**), respectively. *p*_cr1_ = 29 and *p*_cr2_ = 49. (**d**) Propagation of an irregular beam with *γ* = 1.08. *γ* = 3, *d* = 3 in (**a**–**c**); *κ* = 50, *η* ∈ [−25, 25], and *ξ* = 100 in (**a**–**d**). (**e**) Critical depths versus *γ* for complex beams. (**f**) The central position *η*_*c*_ (top) and integral form-factor *χ* (bottom) versus *ξ*. *α* = 1.08 in all the panels.

One natural question arises from the above discussions: Can the conclusions be applied to other forms of light fields? To address this issue, we consider the evolution of modulated Gaussian beams described by Eq. (2) with *γ* ≠ 0. Figure 4(a–c) show clearly that the bound state can also penetrate, be transmitted/reflected, and be totally reflected in shallow, moderate, and deep potentials, respectively. The modulation of a Gaussian beam does not influence its propagation behaviour.

In fact, we numerically examine the evolution of many forms of light fields and find that similar beam propagation management can be exerted on a beam *with arbitrary distribution*, see, e.g., Fig. 4(d), where the input is expressed as ${\rm{\Psi }}=\exp (-{x}^{2}/9)\cos \,[(\sqrt{5}-1)x]\,\tanh \,[x-(\sqrt{5}-1)/2]\exp (i\kappa x)$. This property is fundamentally important in the practical applications. It also gives helpful hints for the understanding of the fractional effects in other fields.

The critical potential depths are invariant with the growth of modulation frequency *γ* [Fig. 4(e)]. They are equal to the critical depths of the Gaussian beam shown in Fig. 3(a). The central coordinate *η*_*c*_ and the integral form-factor *χ* demonstrate that complex beams in deep potentials also follow a zigzag path and are well localized after a large distance [Fig. 4(f)].

### Propagation of beams in adjusted potentials

**Figure 5:**
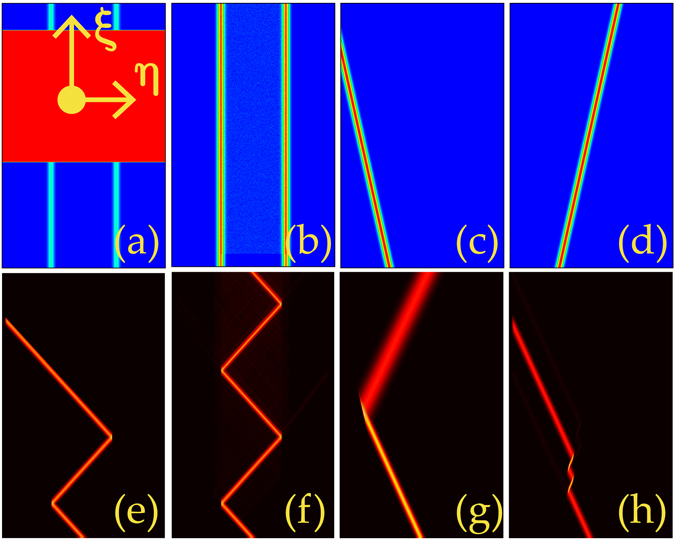
Adjusted potentials (**a**–**d**) and the corresponding novel propagation dynamics (**e**–**h**). (**a**) A block-shaped obstacle with *p* = 180 placed at *ξ* ∈ [40,90]. (**b**) A strong disordered noise with |*σ*_noise_| = 7 added between the potential wells in the scope *ξ* ∈ [5,100]. (**c**,**d**) Examples of inclined Gaussian waveguides with angles equalling ±*π*/9. *p* = 60, *η* ∈ [−25, 25] in all the panels. *ξ* = 100 in (**a**–**h**) and *κ* = 50 in (**e**–**h**).

Next, we address some interesting results when the deep potential is adjusted. A block-shaped obstacle with depth larger than the corresponding *p*_cr2_ is placed on the light path [Fig. 5(a)]. When the original zigzag-propagation beam enters the obstacle, it feels a homogeneous refractive index and propagates straightly without distortions [Fig. 5(e)]. The high-depth obstacle, thus, can be utilized to output the beam trapped in the region between two potentials.

If one adds strong disordered noises with a variance *σ*_noise_ into the region between two waveguides [Fig. 5(b)], the original total reflection remains unchanged provided that |*σ*_noise_| < *p* − *p*_cr2_ [Fig. 5(f)]. As can be explained in physics that beams do not feel the existence of relatively weak noise. Thus, the system we suggested has a high capacity of resisting disturbance.

An expansion is that one can insert a block-shaped waveguide with suitable depth and width into the region between two waveguides to realize the transition between three regimes of beam propagation. This design makes the experimental realization of total transmission, partial transmission/reflection, and total reflection very simple. It is convenient to fabricate a double-barrier potential with a fixed deep depth and some block-shaped waveguides with different depths. By inserting the block-shaped waveguides into the double-barrier structure, one can easily obtain an effective double-barrier potential with required depth.

When the waveguide with large *p* is placed obliquely [Fig. 5(c)], the total reflection still takes place. Yet, after the reflection, the beam becomes broader and propagates without obvious distortions as well [Fig. 5(g)]. It means that beam reshaping can be realized by the suitable placement of waveguide. If the waveguide is inclined towards the other direction [Fig. 5(d)], a different novel phenomenon occurs. At the same potential depth, the original total reflection becomes a total transmission [Fig. 5(h)]. Specifically, the beam is transiently trapped in the waveguide, afterwards, it escapes and propagates straightly. In this process, besides the central beam carrying major energy, there are several output beams with low energy shifting from the main beam along *ξ* direction. These beams are too weak to be distinguished in the electronic plot. The longitudinal displacement between the input and transmitted beams is similar to but different from the classical Goos-Hänchen shift taking place in the total internal reflection in normal or negatively refractive media^36^.

## Theoretical analysis

**Figure 6:**
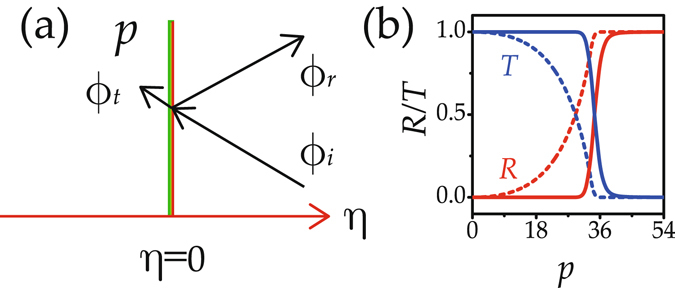
Theoretical analysis scheme and results. (**a**) Schematic of an equivalent setup with a narrow step potential. (**b**) Theoretical (dashed) and numerical [solid and shown in Fig. 3(b)] reflectivity and transmissivity versus potential depth.

To better understand the physics of the above results, it is helpful to conduct a rough theoretical analysis on the system described by Eq. (1). For narrow potentials, the interactions between beams and potentials are mainly determined by the potential depth. For simplicity, we consider a scheme shown in Fig. 6(a), where the narrow Gaussian potential is replaced by a square thin potential. The right interface of the potential is shifted to *η* = 0. These simplifications allow one to grasp the main features of beam dynamics for the adjustable potential depth.

We consider Ψ in its plane-wave solution ${\rm{\Psi }}(\eta ,\xi )=\varphi (\eta )\exp (-i{b}_{\alpha }\xi )$. Here, *b*_*α*_ is the propagation constant relating to the Lévy index. The beam profile is assumed as $\varphi (\eta )={A}_{m}\exp (-{\eta }^{2}/{d}_{m}^{2})\cos (\gamma \eta )\exp (-i{k}_{m}\eta )$, where *k* is the transverse wavenumber and *m* = *i*, *t*, *r* stand for the incident, transmitted, and reflected waves, respectively. Substitute the ansatz into Eq. (1) and remove the common term exp(−*ib*_*α*_ *ξ*) from both sides, one obtains,

3 $${b}_{\alpha }\varphi (\eta )=\frac{1}{2}{(-\frac{{\partial }^{2}}{\partial {\eta }^{2}})}^{\alpha /2}\varphi (\eta )+p\varphi (\eta ).$$

Substituting the incident beam into Eq. (3) and setting *p* = 0, we get an equation describing the beam in the region *η* > 0. By taking Fourier transform, the equation in the spectral space can be written as,

4 $${b}_{\alpha }{\hat{\varphi }}_{i}=\frac{1}{2}{|{k}_{i}|}^{\alpha }{\hat{\varphi }}_{i}.$$

From Eq. (4), we derive the relationship between the propagation *b*_*α*_ and wavenumber *k*_*i*_, i, e., *k*_*i*_ = (2*b*_*α*_)^1/*α*^.

When an incident beam encounters a barrier, it is reflected. According to the law of reflection, it is easily to obtain *k*_*r*_ = −*k*_*i*_. Yet, the amplitude *A*_*r*_ and width *d*_*r*_ will be changed. The total light field in the region *η* > 0 is given by the sum of the incident and reflected parts, i.e., $\varphi (\eta )={A}_{i}\exp (-{\eta }^{2}/{d}_{i}^{2})\cos (\gamma \eta )$ $\exp (-i{k}_{i}\eta )+{A}_{r}\exp (-{\eta }^{2}/{d}_{r}^{2})\cos (\gamma \eta )\exp (i{k}_{i}\eta )$.

Substituting the expression of the transmitted beam into Eq. (3) and taking Fourier transform on the derived equation, one obtains,

5 $${b}_{\alpha }{\hat{\varphi }}_{t}=\frac{1}{2}{|{k}_{t}|}^{\alpha }{\hat{\varphi }}_{t}+p{\hat{\varphi }}_{t}.$$

Obviously, *k*_*t*_ satisfies the condition *k*_*t*_ = (2*b*_*α*_ − 2*p*)^1/*α*^.

At *η* = 0, the light fields satisfy the following boundary conditions,

6 $$(\begin{array}{c}{\varphi }_{i}(0)+{\varphi }_{r}(0)={\varphi }_{t}(0),\\ {\varphi }_{i}^{^{\prime} }(0)+{\varphi }_{r}^{^{\prime} }(0)={\varphi }_{t}^{^{\prime} }(0).\end{array}$$

Assuming *d*_*i*_ ≈ *d*_*r*_ ≈ *d*_*t*_, we obtain,

7 $$(\begin{array}{c}{A}_{i}+{A}_{r}\,={A}_{t},\\ ({A}_{i}-{A}_{r}){k}_{i}={A}_{t}{k}_{t}.\end{array}$$

The amplitude of the reflected and transmitted light fields can be determined by solving Eq. (7), namely,

8 $$(\begin{array}{rcl}{A}_{r} & = & ({k}_{i}-{k}_{t}){A}_{i}/({k}_{i}+{k}_{t}),\\ {A}_{t} & = & 2{k}_{i}{A}_{i}/({k}_{i}+{k}_{t}).\end{array}$$

From Eq. (8), we derive the reflectivity and transmissivity as:

9 $$\begin{array}{rcl}R & = & \frac{{|{A}_{r}|}^{2}}{{|{A}_{i}|}^{2}}=\frac{{({k}_{i}-{k}_{t})}^{2}}{{({k}_{i}+{k}_{t})}^{2}}=\frac{{[{(2{b}_{\alpha })}^{1/\alpha }-{(2{b}_{\alpha }-2p)}^{1/\alpha }]}^{2}}{{[{(2{b}_{\alpha })}^{1/\alpha }+{(2{b}_{\alpha }-2p)}^{1/\alpha }]}^{2}}\end{array}$$

and *T* = 1 − *R*.

The results are shown in Fig. 6(b). From the theoretical analysis, we qualitatively find that the transmission, partial transmission/reflection, and total reflection of a beam occur for small, modulate, and large potential depths, respectively. Another notation is that the transmissivity is independent of the forms of input beams, which indicates that beams with arbitrary distribution obey the same propagation law. An representative example is the evolution of complex beams shown in Fig. 4.

## Conclusions

To summary, we investigated the propagation of optical beams in the FSE with a double-barrier potential. By adjusting the potential depth, one can easily realize the transmission, partial transmission/reflection, and total reflection of approximate diffraction-less beams. Complex beams exhibit the similar propagation dynamics. We also studied the evolution of beams in adjusted potentials, e.g., block obstacle, disorder-defective pathway, and inclined waveguide. Some intriguing properties, including self-healing, reshaping and Goos-Hänchen-like shift were revealed. We anticipate that other forms of external potentials may provide more convenient ways for the beam propagation management. Our results give new insights into the fields of beam management and fractional optics and can find many applications in optical switching, routing, communication, etc.
